# Pulmonary bullet embolism – a safe treatment strategy of a potentially fatal injury: a case report

**DOI:** 10.1186/1754-9493-3-12

**Published:** 2009-06-19

**Authors:** Ali M Hassan, Roger S Cooley, Thomas J Papadimos, John J Fath, Thomas A Schwann, Haitham Elsamaloty

**Affiliations:** 1 Department of Anesthesiology, University of Toledo, College of Medicine, 3000 Arlington Avenue, Toledo, Ohio 43614, USA; 2 Department of Surgery, University of Toledo, College of Medicine, 3000 Arlington Avenue, Toledo, Ohio 43614, USA; 3 Department of Radiology, University of Toledo, College of Medicine, 3000 Arlington Avenue, Toledo, Ohio 43614, USA

## Abstract

**Background:**

Vascular embolization of a projectile discharged from a weapon is a rare event. In this report a hunter's errant gunshot struck a farmer in the left chest.

**Case report:**

The projectile was lodged between the apex of the heart and the diaphragm. The patient was treated non-operatively and was discharged home only to return to the emergency department with chest pain and subsequent identification of the projectile in the left inferior pulmonary vein. Operative management consisted of a median sternotomy, cardiopulmonary bypass, and a pulmonary venectomy.

**Conclusion:**

He was subsequently discharged home and recovered uneventfully.

## Background

Embolism of projectiles is a rare event, in the Vietnam War this occurred in 0.3% of 7500 casualties [1]. There are fewer than 200 cases in the literature since 1900 [2]. This includes all known data from both World Wars and all conflicts in the last century [3-6]. The mortality rate for penetrating gunshot wounds to the chest varies between 14.3% and 36.8% [7]. A missile embolus to the right heart and pulmonary bed after penetrating chest trauma is uncommon [6,8,9], and such an embolus to the left ventricle is even more rare [9-13]. Embedded missiles in the left heart may be observed if they are asymptomatic [14], but if not, then there is a risk of embolization or thrombus and must therefore be removed [11,14,15]. A pulmonary embolus is, indeed, a rare event, and the majority of such patients will be without symptoms, but cough, dyspnea, chest pain, and/or hemoptysis may occur [14-17]. Here we report a unique case of unexplained migration of a pulmonary projectile into the left inferior pulmonary vein. This occurred without clinically apparent intervening damage to the pericardium, heart, lung or vascular structures.

## Case report

**Figure 1:**
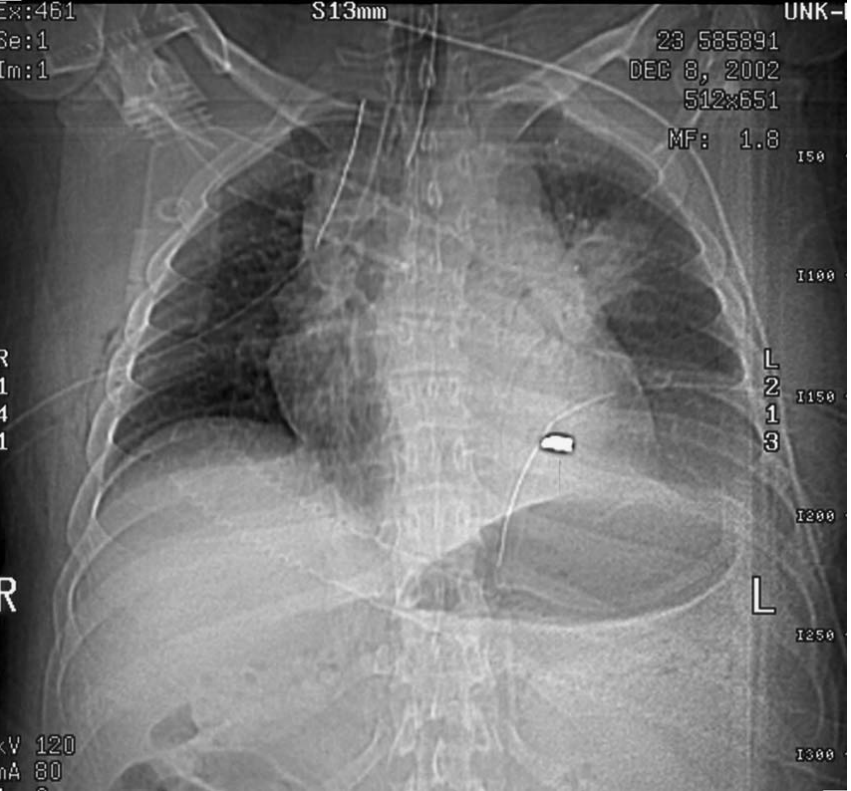
**Scout film of chest with bullet evident in left chest**.

**Figure 2:**
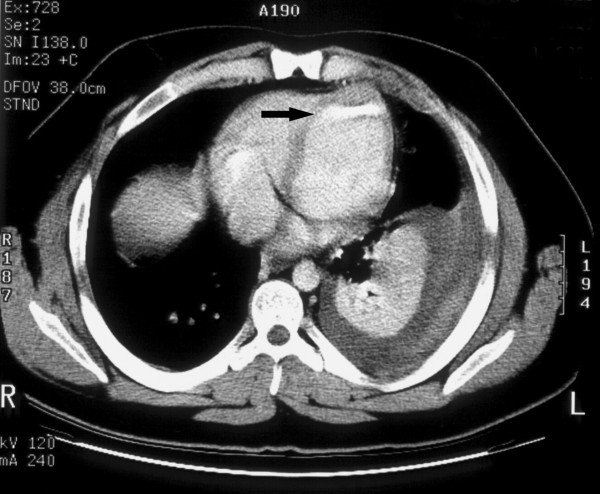
**The initial computerized tomography scan demonstrating the bullet position between the heart apex and diaphragm (see arrow)**.

While working in his field, a 23 year old farmer sustained a gunshot wound to his left upper chest secondary to a hunter's errant shot (a 410 slug, 2.5 inches in length). The projectile entered the left anterior chest between the second and third ribs. Emergency medical services were immediately called. They arrived promptly and found the patient to be hypotensive with a systolic blood pressure of 70 mmHg and with diminished breath sounds over the left lung field. The emergency personnel treated a presumed pneumothorax with placement of a large bore 14-gauge catheter into the upper left chest. The patient's hemodynamics and breath sounds improved. The patient arrived at The University of Toledo Medical Center Emergency Department (ED) via life flight helicopter with two large bore peripheral intravenous lines in place. He had received 2 liters of intravenous crystalloid in transit. Examination in the ED showed the patient to be hemodynamically stable with good breath sounds and no hemoptysis. The patient presented with a Glasgow Coma Scale of 15, a blood pressure of 128/59 mm Hg, a heart rate of 108/minute, respiratory rate of 20/minute, and an oxygen saturation of 97% on room air. The patient related that he had no allergies, no significant prior medical or surgical history, and was otherwise healthy. Upon examination an entry wound was evident in the left anterior chest and no exit wound was noted. A chest x-ray was ordered, and demonstrated a 15% left sided pneumothorax. The projectile was located in the inferior portion of the left pleural cavity. Bilateral 36 French chest tubes were placed (the right chest tube was prophylactic), and the patient was intubated with an 8.0 tracheal tube, sedated with propofol, and taken for computerized tomography (CT) scan with contrast. The CT scan confirmed the left pneumothorax, with appropriate placement of the bilateral chest tubes and the endotracheal tube. The CT scan also identified the projectile path through the left lung parenchyma. The integrity of all major vessels and tracheobronchial segments was confirmed. The projectile was identified between the apex of the heart and the diaphragm (see figures 1, 2).

The patient was admitted to the surgical intensive care unit (SICU), where he remained hemodynamically stable. Serial roentgenograms revealed the projectile remained immobile. He was weaned from the ventilator and extubated on hospital day 2 in the SICU. The right chest tube was removed on day 3, and the left chest tube was removed on day 5. The chest roentgenogram on day 5 showed no recurrent pneumothorax. The patient was discharged on the afternoon of the fifth hospital day with elective surgery planned for the removal of the projectile.

**Figure 3:**
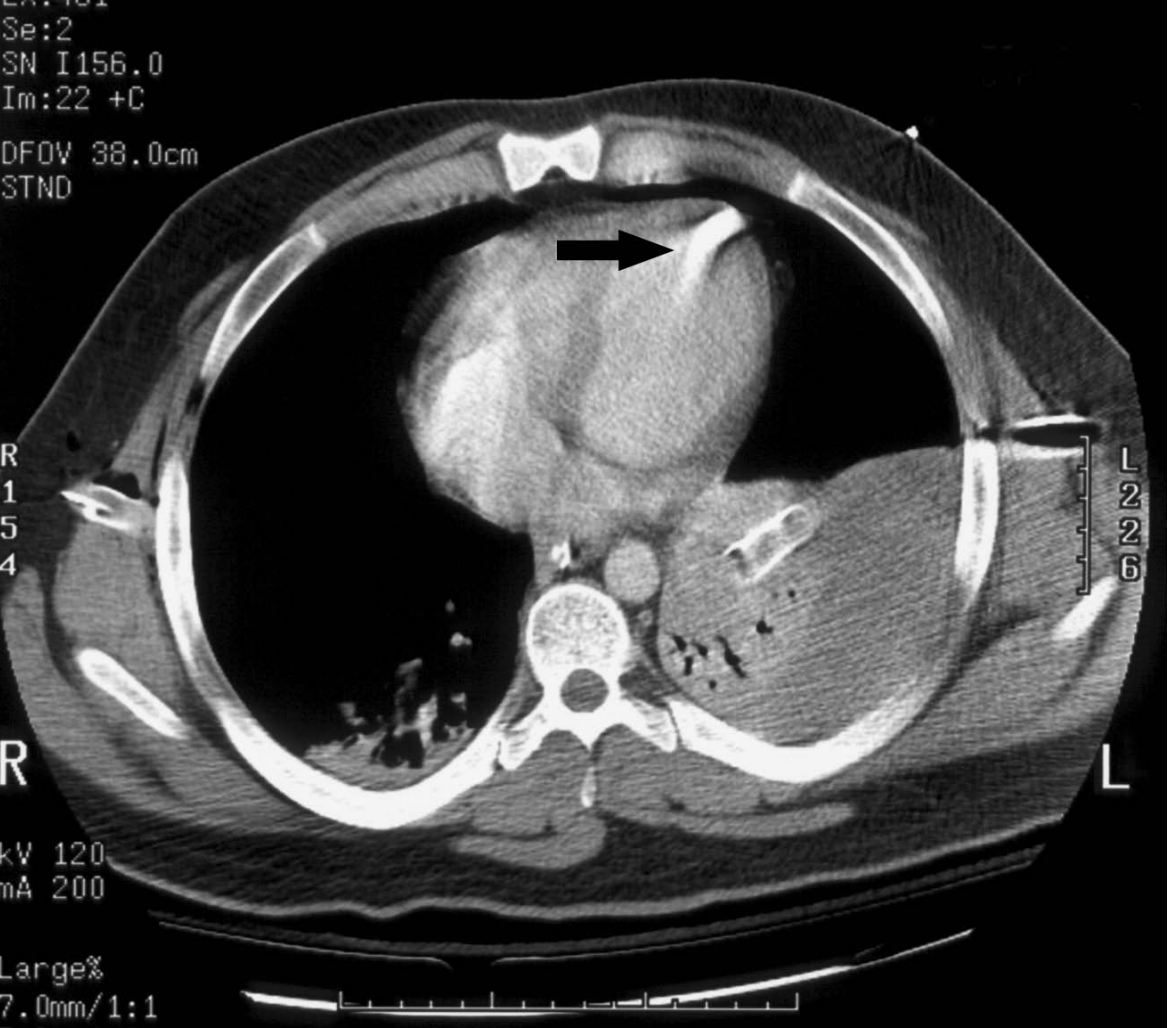
**Repeat computerized tomography scan on readmission indicating the projectile may be in a cardiac chamber, imbedded in the cardiac muscle, or in a pulmonary vein/pulmonary vein branch (see arrow)**.

The patient subsequently returned to the ED on the third post discharge day with complaints of pain at the left chest tube insertion site. He denied any interval hemoptysis or shortness of breath. Chest roentgenogram revealed the projectile had migrated to a position near the left atrium. CT examination demonstrated the projectile was in the heart or, possibly, in a vessel near the heart (see figure 3).

An urgent cardiothoracic intervention was scheduled on the same day. After induction of general anesthesia, an 8.0 single lumen tube was placed. A left endobronchial blocker was placed to facilitate single lung ventilation as required. A right internal jugular introducer was inserted with subsequent insertion of a pulmonary artery catheter. Additionally, a 20 gauge right radial arterial line was placed. Transesophageal echocardiography (TEE) monitoring was used to monitor cardiac function throughout the case. Initial TEE and intraoperative fluoroscopy further confirmed the roentgenographic and CT findings suggesting the projectile was in or near the left atrium. A median sternotomy was performed to explore the left thoracic cavity and revealed that the pericardial sac and heart surfaces were intact. In addition, the major vessels and lung surfaces also appeared to have no defects. Cardiopulmonary bypass was instituted, and exploration of all four cardiac chambers was done via incision of the biatrial groove. No palpable evidence of the projectile was noted at that time. Repeat TEE and fluoroscopy showed the projectile to be in the same position. Re-exploration of the posterior aspect of the heart revealed a palpable object in the left inferior pulmonary vein. A venotomy of the left inferior pulmonary vein was performed and the projectile was removed intact. After careful reinspection of the vasculature and heart, two chest tubes were placed and the thorax was closed. The patient was transferred to the SICU.

A bronchosopy was done on postoperative day 3 and confirmed that the left tracheobronchial tree was intact with no evidence of bleeding. The patient was extubated with subsequent discontinuation of the chest tubes. He recovered uneventfully and was discharged on postoperative day six.

## Discussion

There are two general scenarios for projectiles striking a body: high speed (military) and low speed (civilian) [18,19]. In the case of a high-speed projectile impacting a body, the projectile will enter the body, travel in a straight line and exit the body. Secondary internal damage is due to bone fragments or viscoelastic shock waves disrupting other structures. With a low speed impact, the projectile may not travel in a straight line, and it may not exit the body. Tissue planes, bone, or other structures can deflect a low speed projectile. Any time a projectile enters a body and does not have an exit wound, very careful exploration of the projectile path is warranted. If the projectile is not found along the expected path, clinically or radiographically, embolization of the projectile should be suspected.

When such an embolism does occur it usually involves low speed projectiles [3,4]. Most cases involve arterial embolization (70–75%) versus venous embolization (25%) [5,6]. Antegrade and retrograde projectile migration within the arterial or venous vessels is possible [3]. Projectiles or fragments may enter the cardiovascular system in one of four ways:

1) Directly into one of the cardiac chambers, where it remains within the heart, or enters an outflow tract to the lungs, or the arterial system. Paradoxical migration has been described through a patent foramen ovale [6,20,21].

2) Directly into an artery, and then antegrade to the point of entrapment depending on the projectile size and vessel lumen [22].

3) Directly into a vein, where either antegrade or retrograde embolization is possible. This is dependant on patient position, vessel and projectile size. Retrograde venous embolization usually occurs in the very large veins when the patient is in the upright position, or with coughing, or a valsalva maneuver [3,4,6,22,23].

4) Erosion of the projectile through the pulmonary tissue into a pulmonary vessel; and either to the pulmonary arterial bed, or as in this case, into the pulmonary venous bed with potential to become arterial.

In this case report the patient experienced an antegrade venous embolization of a 410 slug. Up to 70% of patients with venous projectile embolization remain hemodynamically stable and may be asymptomatic [3]. In this case there was also delayed migration of the projectile into the left inferior pulmonary vein. Delayed migration of a retained projectile and other complications such as: organ infarction, septicemia, and thromboembolism has also been demonstrated in up to 25% of patients [24]. The first option for removal of an intravascular projectile embolus is the use of interventional radiology [3]. If this is not feasible, then surgery utilizing intraoperative fluoroscopy is mandatory [3].

Even though the projectile may have eroded from the lung base into a pulmonary vein that drains that segment, we suspect the projectile was actually in the venous circulation initially and its original location was misidentified. If the projectile did erode into a pulmonary vein it would be the first such occurrence ever reported.

We wish to emphasize to our colleagues that vigilance is required in the hemodynamically stable, asymptomatic patient with a retained projectile in the pulmonary bed secondary to a gunshot wound who is discharged home for follow up care and elective surgery. We suggest that when a chest roentgenogram identifies a projectile/missile in the pulmonary bed that CT scanning or angiography be used to confirm its position. If the patient is symptomatic then the projectile should be removed. In the operative theatre transesophageal echocardiography will be of benefit for location of the projectile and for following the function of the heart. Intraoperative fluoroscopy may also be of value. However, in this case palpation of the involved pulmonary vein by the surgeon was the only absolute way to identify the projectile's location. While these missile emboli can be followed clinically in asymptomatic patients, such management remains a point of controversy [1,2,16,25,26].

## Conclusion

Missile emboli that gain access to the pulmonary bed, and eventually the heart, are a rare event. Patients that are asymptomatic may be treated without surgery. Nonetheless, surgical intervention should be seriously considered before releasing the patient from the hospital. A patient exhibiting any symptoms will need an urgent/emergent operative intervention.

## Consent

Consent was received from the patient. A copy of the written consent is available from the editor-in-chief of this journal

## Competing interests

The authors declare that they have no competing interests.

## Authors' contributions

AMH provided the anesthesia for this case, as well as the technical information about the case and aided in the writing of the manuscript. RSC collected and compiled the information, wrote and revised the manuscript. TJP was the main technical adviser that wrote multiple revisions of the manuscript and oversaw the submission of this article. JJF provided detailed technical information about cardiothoracic penetrating trauma and literature information. TAS was the cardiothoracic surgeon of this case who provided technical information and description of the surgical procedure for this case. HE provided clinical and technical radiographic guidance. All authors read and approved the final manuscript.
